# Cardiac Hemangioma: A Rare Tumor Presenting as Postpartum Chest Pain

**DOI:** 10.7759/cureus.44407

**Published:** 2023-08-30

**Authors:** Mihika Shah, Lori P Russo, Daniel Haddad, Joanne Chang, Arthur Okere

**Affiliations:** 1 Anesthesiology, Mount Sinai Morningside/West, New York, USA; 2 Anesthesiology, Hackensack Meridian Ocean University Medical Center, Brick, USA; 3 North American Partners in Anesthesia (NAPA), Hackensack Meridian Ocean University Medical Center, Brick, USA; 4 Anesthesiology, Westchester Medical Center, Valhalla, USA; 5 Obstetrics and Gynecology, Hackensack Meridian Ocean University Medical Center, Brick, USA; 6 Cardiology, Hackensack Meridian Ocean University Medical Center, Brick, USA

**Keywords:** preeclampsia, venous thromboembolism, postpartum chest pain, cardiac tumor in adults, cardiac hemangioma, transesophageal echocardiography (tee)

## Abstract

Pulmonary embolism, peripartum cardiomyopathy, acute myocardial infarction, aortic dissection, anxiety, and gastroesophageal reflux disease are known causes of chest pain during the peripartum period. A cardiac tumor is a rare cause of chest pain during this time period. While cardiac myxomas during pregnancy have been reported, cardiac hemangiomas are exceptionally rare. To the best of our knowledge, there are no existing case reports regarding cardiac hemangiomas in either pregnant or postpartum patients. Here, we present a 23-year-old female who presented with visual changes, headache, and midsternal pain and was subsequently found to have a cardiac hemangioma.

## Introduction

The postpartum period, also known as the fourth trimester of pregnancy, is a period of time when patients can be susceptible to complications similar to those seen during pregnancy. These include seizures, stroke, hemorrhage, and pulmonary embolism (PE) [1].

Patients may present with chest pain, which warrants further evaluation. Most commonly, the origins of postpartum chest pain are musculoskeletal, gastrointestinal, cardiac, or psychological. Chest pain may be a presenting symptom of postpartum anxiety, depression, and/or panic disorder. Postpartum depression may occur in up to 50% of all obstetric patients with anxiety and panic disorder being severe manifestations [1-2]. Gastroesophageal reflux disease (GERD) is also a common cause of chest pain. During pregnancy, the uterus increases pressure on the gastrointestinal tract and stomach resulting in reflux. Postpartum GERD can be exacerbated resulting in symptoms such as heartburn and chest pain [1]. Cholelithiasis is another common cause of upper abdominal pain with referred shoulder and chest pain. Increased cholesterol saturation of bile and decreased motility of the gallbladder during pregnancy and postpartum results in an increased risk of cholelithiasis. Prepregnancy obesity and multiparity are risk factors [3]. More serious causes of chest pain in the postpartum period include acute myocardial infarction (MI), aortic dissection, and PE. Although the incidence is low overall, there is an increased risk of acute MI and aortic dissection in pregnant patients when compared to age-matched nonpregnant patients, particularly in pregnant and postpartum patients with a history of bicuspid aortic valve or connective tissue disorders. Hypertension, especially, is a risk factor for aortic dissection and patients like ours can present with preeclampsia postpartum [1,4,5].

PE accounts for 9.2% of all pregnancy-related deaths in the United States [6]. Venous thromboembolism, a well-established risk factor, occurs in approximately one out of 1600 pregnant women [7]. PE should be suspected in a postpartum woman presenting with sudden dyspnea, pleuritic pain, chest pain, and/or hemoptysis. Peripartum cardiomyopathy (PPCM) should also have a high index of suspicion in a peripartum woman presenting with dyspnea or chest pain as initial symptoms of shortness of breath, pedal edema, and nonspecific fatigue are commonly seen in late pregnancy. 

In pregnant and peripartum patients presenting with new-onset hypertension, preeclampsia should always be considered due to the rapid progression to severe features. When new-onset hypertension is associated with significant proteinuria in combination with visual changes, persistent headaches, and/or other signs of end-organ dysfunction, severe preeclampsia is likely. Differential diagnoses to consider for severe preeclampsia include HELLP (hemolysis, elevated liver enzymes, low platelets) syndrome, acute fatty liver of pregnancy, thrombotic microangiopathy (such as thrombotic thrombocytopenic purpura, systemic lupus erythematosus, and antiphospholipid syndrome) [8]. Late or delayed-onset postpartum preeclampsia is an atypical preeclampsia characterized by the onset of signs and symptoms leading to readmission more than two days but less than six weeks after delivery [9]. Risk factors for late postpartum preeclampsia have been shown to be similar to that of typical preeclampsia including age ≥40 years, Black race, Latino ethnicity, final pregnancy body mass index of ≥30 kg/m^2^, and gestational diabetes mellitus [9-11]. In a retrospective cohort study with 152 patients diagnosed with late postpartum preeclampsia, 63.2% had no history of hypertensive disease in the previous pregnancy, 18.4% had preeclampsia, 9.2% had chronic hypertension, 4.6% had gestational hypertension, and 4.6% had preeclampsia superimposed upon chronic hypertension during the peripartum period. Of those patients diagnosed with late postpartum preeclampsia, 14.5% went on to develop postpartum eclampsia, and 70% of those patients presented with a headache as their chief complaint [11]. 

While the literature contains cases and studies pertaining to postpartum chest pain secondary to musculoskeletal and gastrointestinal causes as well as cardiopulmonary-related ones such as acute MI, aortic dissection, and PE, to the best of our knowledge, there does not exist a case report of a cardiac hemangioma (CH) presenting as postpartum chest pain. CHs are very rare, representing less than 3% of all diagnosed cardiac tumors. Additionally, they are more commonly seen in males and in those in their fifth decade of life [12]. Here, we present a novel case of a female in her third decade of life whose presenting symptom was postpartum chest pain who was diagnosed with a CH during the workup for a PE.

## Case presentation

A 23-year-old G3P0111 with a history of anxiety and ovarian cysts arrived at our emergency room at 35 weeks and six days of gestation with contractions occurring four to five minutes apart. She was admitted to the labor and delivery unit of our hospital for the evaluation of preterm labor. For the purposes of this case report, we will refer to our hospital as “Hospital A.” Her blood pressure when seen by the obstetric team was 175/95 mmHg with a hemoglobin of 8.0 g/dL. Several hours after admission, the patient requested labor analgesia, and an epidural was placed with resolution of her pain. Approximately four hours after the placement of the epidural, a healthy female baby was born. Later in the evening, the patient reported passing a large blood clot associated with dizziness when she used the restroom. A repeat hemoglobin was obtained and came back at 7.0 g/dL The decision was made to observe her without transfusing blood. Blood pressure measurements remained in the 140-150 mmHg systolic range for the hospitalization but otherwise, her stay was unremarkable and she was discharged home on postpartum day two. 

**Figure 1:**
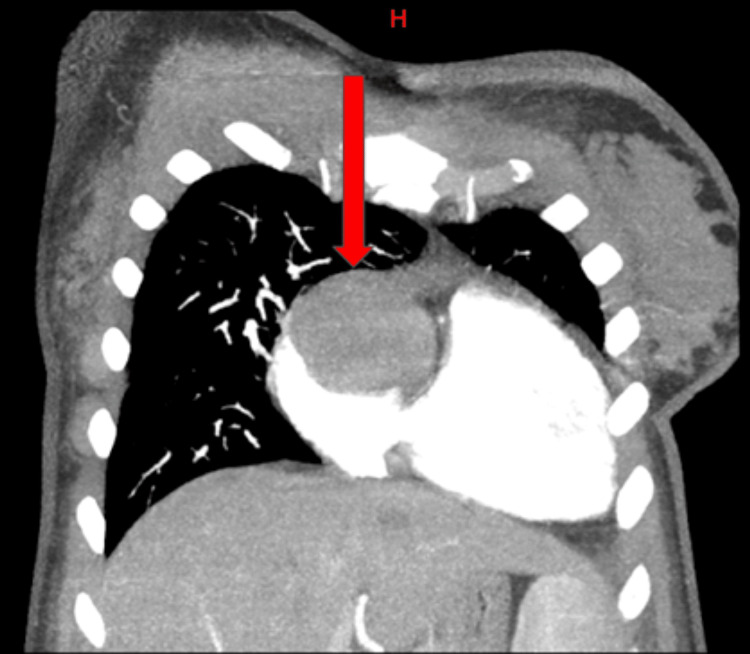
Computed tomography angiography of intracardiac mass H: head

On postpartum day 5, the patient reported visual changes, headache, and dull, throbbing, midsternal chest pain for which she was referred to the emergency department for direct admission under the care of the obstetrician to start a magnesium infusion for presumed severe pre-eclampsia. Her blood pressure was 159/88 mmHg on arrival. Given the patient’s complaint of chest pain, the decision was made to evaluate this further in the emergency room. An electrocardiogram (EKG) was performed and was unremarkable. A troponin test showed 0.07 ng/dL and brain natriuretic peptide level was 1,168 pg/dL. The urine protein level was 100 mg/dL (normal reference range is negative). A chest x-ray was notable for moderate airspace disease at the bilateral mid to lower lung fields. Given the patient’s recent pregnancy, associated inherent hypercoagulability, and chest pain, the patient was sent for a computed tomography angiography (CTA) of the chest for the evaluation of a PE. The scan was negative for a PE but did yield moderate bilateral pleural effusions and a solid mass of 6.5 cm, probably intracardiac and located within the right atrium (Figure 1).

The cardiology team was consulted. Transesophageal echocardiography (TEE) was performed showing a 7.2 x 6.8 cm non-mobile, encapsulated mass in the right atrium with motion that appeared to be congruent with the cardiac wall and did not completely obstruct blood flow. The mass did, however, compromise the tricuspid valve (Video 1). The patient was transferred to the intensive care unit while she awaited transfer to our tertiary care center, "Hospital B," with cardiac surgery services.

**Video 1:** Transesophageal echocardiography showing a large right atrial mass

The following day, postpartum day 6, the patient was scheduled for resection of her right atrial mass via open heart surgery at Hospital B. It was noted that the right atrium was firm to the touch over the anterior free wall extending to the superior vena cava, down to the junction of the right atrium and aortic root, and along the junction of the right ventricular free wall in the area of the right coronary artery. In addition, the patient had friable tissue and the decision was made to obtain a sample for a frozen section using a needle biopsy. An initial pathology report showed spindle cells but the final diagnosis was pending permanent section results. Because of the complex nature of the mass, a joint decision was made between the surgical team, the cardiologist, the patient, and her family to transfer the patient to a larger academic referral hospital, which we will refer to as "Hospital C."

Once transferred to Hospital C, the patient was started on a nicardipine infusion for blood pressure control. A transthoracic echocardiogram showed normal biventricular function, mild enlargement of the left atrium, and a large mass in the right atrium. Cardiac magnetic resonance imaging (MRI) yielded a large hypervascular mass centered at the atrioventricular groove, either extending into or causing significant compression of the right atrium. The pathology read from Hospital B also stated that the findings were concerning for a cardiac angiosarcoma with the given history of spindle cells on recent pathology. The slides from Hospital B where the biopsy was taken were requested by Hospital C. The patient was discharged home on postpartum day 13 (post-biopsy day 9) with a plan to have Hospital C review the slides from Hospital B, to obtain a positron emission tomography (PET) scan and for outpatient follow-up with oncology and cardiothoracic surgery services.

The PET scan revealed redemonstration of a mild fluorodeoxyglucose (FDG)-avid right atrial mass consistent with the known malignancy (7.0 x 6.4 cm). There was heterogeneous FDG uptake in the anterior mediastinum with a subsequent photopenic area, likely related to the biopsy. A review of the pathology slides from Hospital B by Hospital C revealed a vascular mass with infiltration into the tissue of the myocardium with associated degenerative changes in the myocardium along with scarring. This was felt to be a benign hemangioma. The patient met with oncology and it was determined that because of the pathology results, chemotherapy was not warranted. Surgical resection was recommended.

The patient underwent excision of the right atrial mass with patch reconstruction at Hospital C on postpartum day 39. She was admitted to the intensive care unit immediately following surgery. On postoperative day 1, she was transferred to the step-down unit. One unit of packed red blood cells was given for a low hemoglobin level on postoperative day two. She received physical therapy throughout her postoperative stay. Chest tubes were removed and the patient was discharged home on postoperative day 4 after an unremarkable postoperative course and resolution of her dull, throbbing, midsternal chest pain.

## Discussion

CHs are rare to see in clinical practice, representing only 2.8% of cardiac tumors diagnosed. CHs, like all hemangiomas, are proliferations of benign endothelial cells that line blood vessels. Hemangiomas are divided into three types depending on the type of blood vessel involved and include cavernous, capillary, and arteriovenous hemangiomas. They may involve the pericardium, myocardium, or endocardium. These tumors are most commonly seen in those in the fifth decade of life with a slight predilection for males. Additionally, 21% of patients diagnosed with CH have a history of hypertension [12]. While CHs do not have the capacity to metastasize and are considered slow-growing, they can cause catastrophic events such as arrhythmias related to the invasion of cardiac conduction tissue, stroke, syncope, and even sudden cardiac death. CHs may also develop in the fetal heart [12].

In an analysis by Li et al. of over 200 cases of CHs, symptoms included reduced exercise tolerance (24.5%), shortness of breath (22.5%), chest pain/discomfort (22.5%), palpitations (19.9%), syncope (9.3%), edema of the lower extremities (7.3%), angina (7.3%), and stroke (4.0%). Of the patients in the analysis, 36% had a heart murmur on exam. CHs have been noted in all chambers of the heart; however, the right atrium was the most common location. They vary in size from 0.5 cm to 14 cm with an average size of 4.48 cm [12]. Our 23-year-old female patient had a 7.0 cm x 6.4 cm hemangioma in her right atrium.

CHs are exceedingly rare in pregnancy and are more frequently diagnosed in utero in fetuses. To the best of our knowledge, there are no case reports of peripartum CHs to date. In fact, one of the most common cardiac tumors in pregnancy and the peripartum period are myxomas. They occur in all age groups but typically occur between the third and sixth decades of life with a predominance in females. A majority of the myxomas occur sporadically with infrequent reports of familial myxomas. The tumors usually develop in the atria; 75% originate in the left atrium and 15-20% in the right atrium. They are usually endocardial in origin and can range in size from 1 cm to 15 cm with most ranging from 5 cm to 6 cm [13]. 

Our patient was diagnosed in the postpartum period with chest pain although she did arrive at the emergency room in preterm labor with gestational hypertension. It is unclear whether this patient’s generalized anxiety disorder could have masked these symptoms of circulatory compromise, such as palpitations and tachypnea, commonly seen in CH patients. On postpartum day 5, our patient presented to the emergency department with visual changes, headache, and chest pain as well as a blood pressure of 159/88 mmHg. Notably, visual changes are not a commonly reported symptom of CH [12]. This presentation was initially suggestive of a possible PE or late postpartum preeclampsia, urgent pathologies more commonly found in the peripartum period, and which require prompt management [9]. A CTA was ordered to rule out a PE, which revealed our patient’s intracardiac tumor. 

Initial workup for CH includes chest x-rays, EKG, and coronary angiograms. In patients who had chest x-rays done during their workup for CH, 44.6% had abnormal cardiac silhouettes. EKG were most likely to show non-specific ST changes with the second most common EKG result yielding a normal study. Other EKG results include atrioventricular blocks and low-voltage tracings. Additional imaging techniques such as MRI and CT can be used to further evaluate the characteristics of the hemangiomas, including size and location [12]. 

Complications from untreated CHs include embolism, rupture, and sudden death. For this reason, surgical excision is indicated in symptomatic patients despite controversial data regarding the most effective treatment. Some experts recommend conservative medical treatment with vascular endothelial growth factor antagonists, beta-blockers, corticosteroids, interferons, and radiotherapy. Of these, radiotherapy, corticosteroids, and beta blockers were the only effective treatments in scattered case reports. However, there is no cohesive evidence for the most effective treatment. In fact, the prognosis of CH is unfavorable despite treatment. Per Li et al., postoperative long-term adverse effects were observed and included sudden death, refractory ventricular tachycardia, complete atrioventricular block, recurrent pleural effusion, angiosarcoma, and tumor regrowth [12]. Overall, despite the lack of definitive evidence-based studies for treatment options, surgical resection is the recommended treatment with conservative medical management for unresectable tumors. 

## Conclusions

Postpartum chest pain, though more commonly present due to musculoskeletal, gastrointestinal, or psychological reasons, can also be due to uncommon reasons such as cardiac tumors. Cardiac tumors are rare causes of chest pain during pregnancy, with no known cases of CHs in the postpartum period noted in the literature. They are more common in males and in the fifth decade of life. Our unique case report highlights a 23-year-old female who presented with visual changes, headache, and dull, throbbing, and midsternal chest pain on postpartum day 5 and who was found to have a CH. At the initial postpartum visit, the patient underwent a CTA which revealed a cardiac mass; preliminary biopsy results revealed spindle cells. A PET scan revealed the known malignancy in the right atrium and a reanalysis of the pathology slides determined a benign CH. Surgical resection was recommended and the patient underwent excision on postpartum day 39. She was discharged on postoperative day 4. 

This case demonstrates that chest pain in a postpartum patient can be easily misdiagnosed as PE, preeclampsia, or anxiety, especially when other symptoms are present. Therefore, chest pain should be thoroughly explored in postpartum patients. Workup includes, but is not limited to, chest x-rays, EKGs, and CTs. This is especially important when patients have recurrent or unresolved symptoms. This case report contributes to the limited existing literature on peripartum CH. 
